# Supplementary material for: Feature-specific neural reactivation during episodic memory
Source: Nat Commun. 2020 Apr 23;11:1945. doi: 10.1038/s41467-020-15763-2 (PMC7181630; doi:10.1038/s41467-020-15763-2)
Supplement: Supplementary file 1 — Supplementary Information [file 41467_2020_15763_MOESM1_ESM.pdf]

Feature-Specific Neural Reactivation during Episodic Memory

Michael B. Bone[1,2,*], Fahad Ahmad[1], Bradley R. Buchsbaum[1,2]

[1] Rotman Research Institute at Baycrest; Toronto, Ontario, M6A 2E1; Canada
[2] Department of Psychology; University of Toronto; Toronto, Ontario, M5S 1A1; Canada
[*] Lead Contact. Correspondence: michael.bone@mail.utoronto.ca

Supplementary Figures

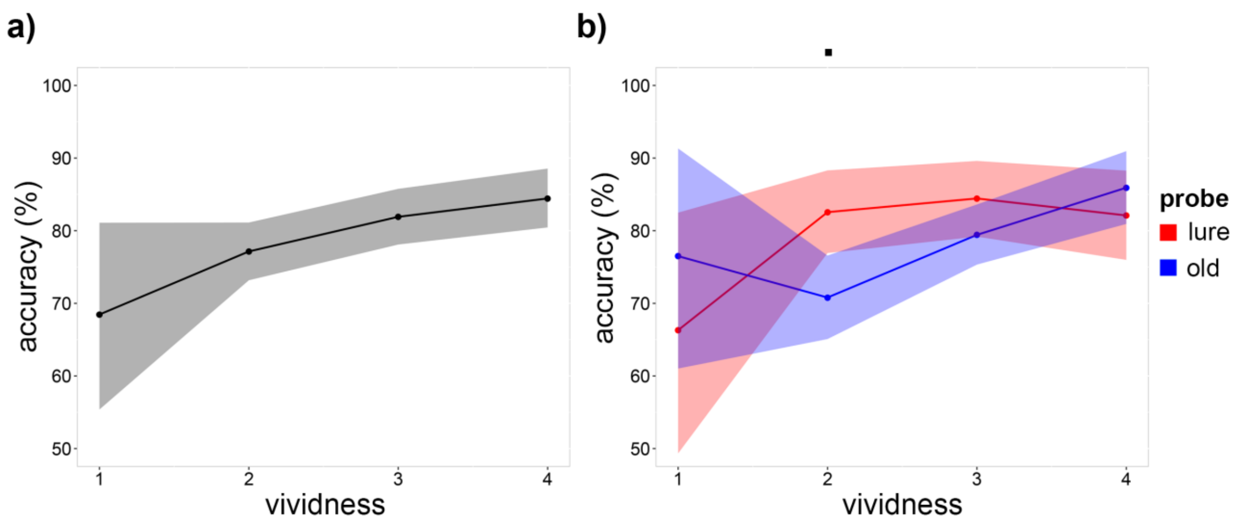

Supplementary Figure 1. Relation Between Vividness and Recognition Accuracy. Average accuracy by vividness rating for a) all trials, and b) "old" and "lure" trials (chance = 50%). To assess the mean, linear mixed-effects (LME) models were used, with accuracy (correct = 1, incorrect = 0) as the dependent variable (DV), and participant and image as crossed random effects (random-intercept only, due to model complexity limitations). Statistical assessments were performed using bootstrap analyses (n = 2406 trials; trials with no vividness response were excluded), calculated with the BootMer function[1] using 2000 samples. Ribbons indicate 90% CIs. The • in b) indicates a marginal difference between old and lure trials, with greater lure-trial accuracy when participants rated the trials' vividness as 2 [p = .060; two-tailed, FDR corrected over vividness ratings]. This finding, in addition to the significant correlation between accuracy and vividness on old (but not new) trials (see the "Relation Between Vividness Ratings and Old/New Task Accuracy" results section of the article), suggests that highly vivid recall impacts old trial accuracy, whereas any recollection beyond "a very small number of visual details" (vividness 1) is sufficient for lure trials. Supporting the latter observation, accuracy for vividness 2-to-4 (pooled together) was significantly greater than accuracy for vividness 1 on lure trials [p = .045; one-tailed].

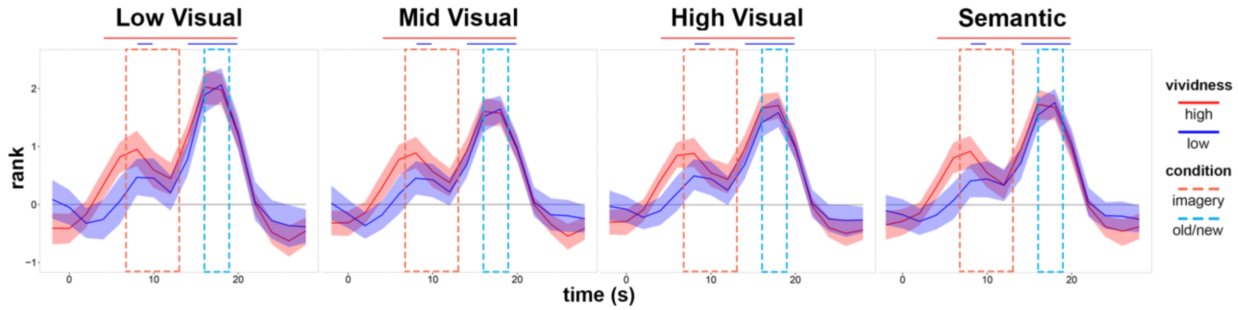

Supplementary Figure 2. Classification Performance Over the Retrieval Period. Whole-cortex classification performance (rank measure) of the cued image divided into low and high vividness trials (for each subject, vividness values were z-scored and trials with values greater than zero were placed in the high vividness group, and all other trials in the low vividness group) over the entire retrieval period. Separate peaks corresponding to the recall and recognition tasks can be clearly distinguished. Ribbons indicate 90% CIs. Lines above the graph indicate time points with classification accuracy greater than chance; $p < .05$, one-tailed, FDR corrected. CIs and p values calculated by bootstrapping (1000 samples) over subjects' mean reactivation values. Task periods offset by 6s to account for hemodynamic delay.

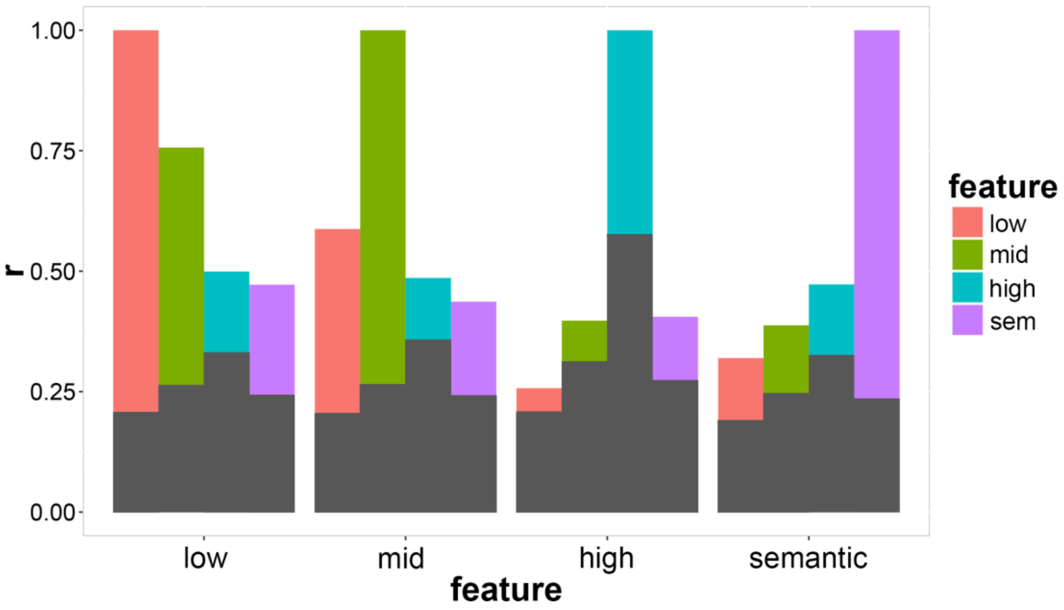

Supplementary Figure 3. Correlation Between Features from Different Layers of a CNN. Grey bars indicate 95% upper bounds of the null distribution (1000 sample permutation). All cross-feature-level correlations were found to be significantly greater than chance, with the correlation magnitude inversely proportional to the distance between the layers. To generate the correlation values, activation vectors (with one value for all 90 image pairs, e.g. 180 images/elements in length) for each of *M* node/features in one layer (represented by the x-axis) were correlated with all *N* node/features in another layer (represented by the legend/color), producing an $M \times N$ correlation matrix. For each row of the correlation matrix, the maximum correlation value was extracted, and the resulting *M* correlation values were averaged (mean).

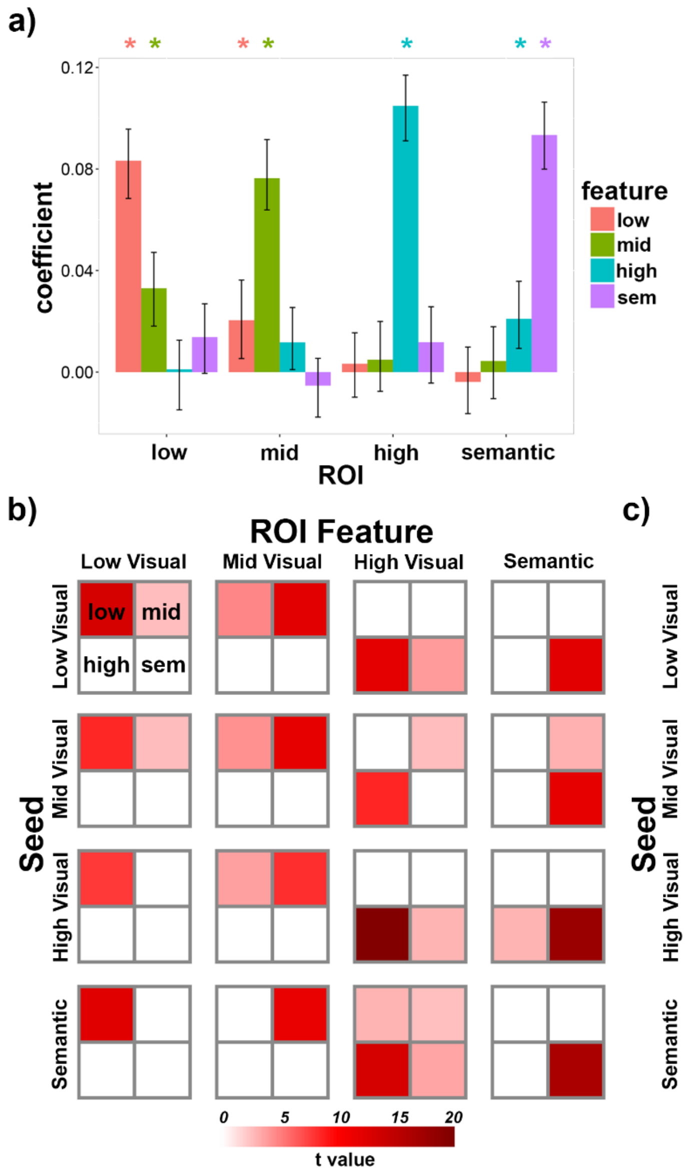

Supplementary Figure 4. Simulated Results for Feature-Specific Informational Connectivity. fMRI data was simulated (200 simulated subjects; see Methods section) and then run through the processing pipeline for FSIC (see Methods section) to validate the approach. ROIs only contain features from the indicated feature-level. a) FSIC results for all combinations of ROI and feature-level, assuming identical trial-by-trial memory accuracy across feature-levels. A separate seed was used for each feature-level, with each containing an equal number of voxels (25) per feature-level. Error bars are 90% CIs; * indicates $p < 0.05$, one-tailed bootstrap, FDR corrected. The similarity of the results to Figure 3b indicates that the feature specificity of FSIC does not depend on the seed disproportionately representing the target feature-level. b) and c) FSIC results for all combinations of seed ROI/feature-level (rows) and target ROI feature-level (columns), assuming b) identical and c) independent trial-by-trial memory accuracy across feature-levels. The squares divided into four sub-squares represent a simulated brain composed

of four target ROIs. Each ROI contains features from one feature-level, as indicated in the top-left corner. t-values are thresholded at $p < 0.05$, one-tailed bootstrap, FDR corrected. Under the cross-feature-level memory dependence assumption, seed selection had little effect on the results, whereas under the independence assumption, significant effects were primarily limited to the diagonal (i.e. when the seed and the target feature correspond).

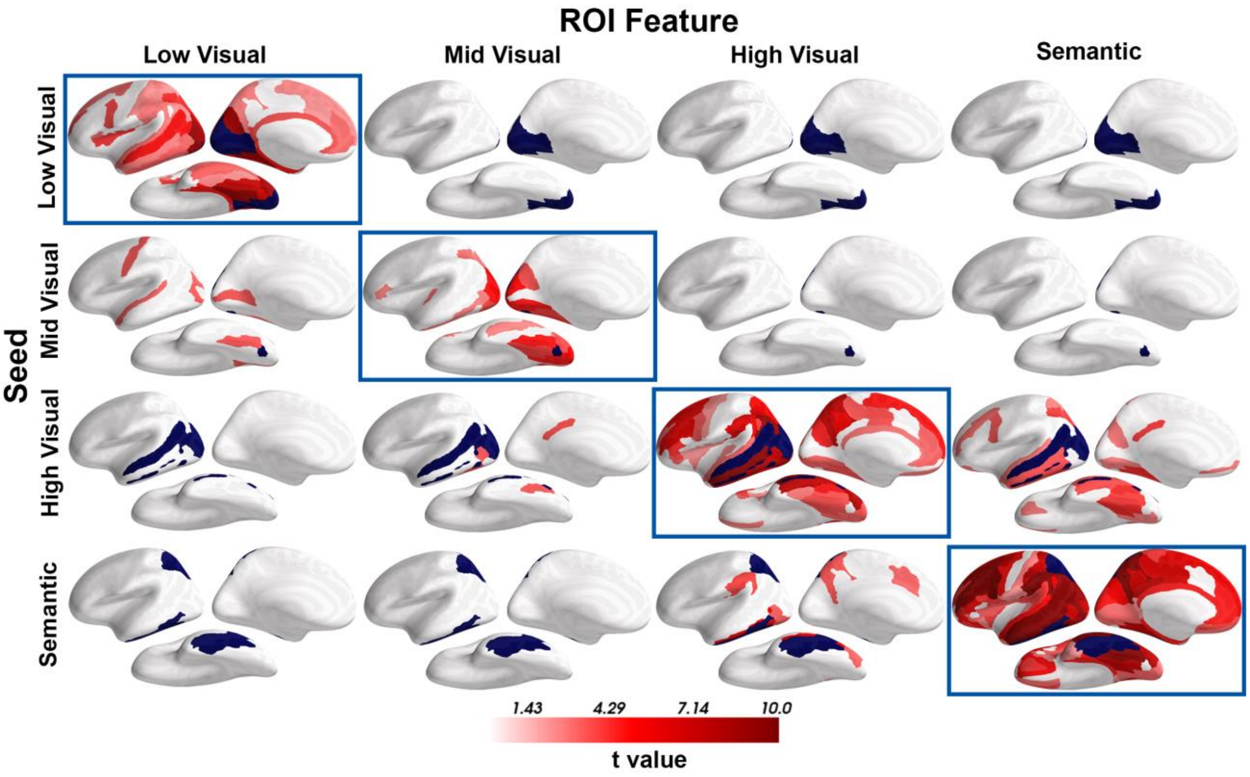

Supplementary Figure 5. Feature-Specific Informational Connectivity during Episodic Recall using All CNN Layers. A recreation of the analysis depicted in Figure 4b with the exception that all sixteen layers from VGG-16 were used instead of layers 2, 7, 13 and 16. The sixteen layers were divided into four groups, with convolutional layers 1-4, 5-9, 10-13 and fully-connected layers 14-16 assigned to the low-, mid-, high-visual and semantic groups, respectively. The reactivation values for each of these layers were then averaged, producing a single reactivation value for each of the four groups for each combination of subject, ROI and trial. FSIC was then performed a manner identical to how the results in Figure 4b were attained. The strong similarity between these results and the original suggests the findings are robust to layer selection.

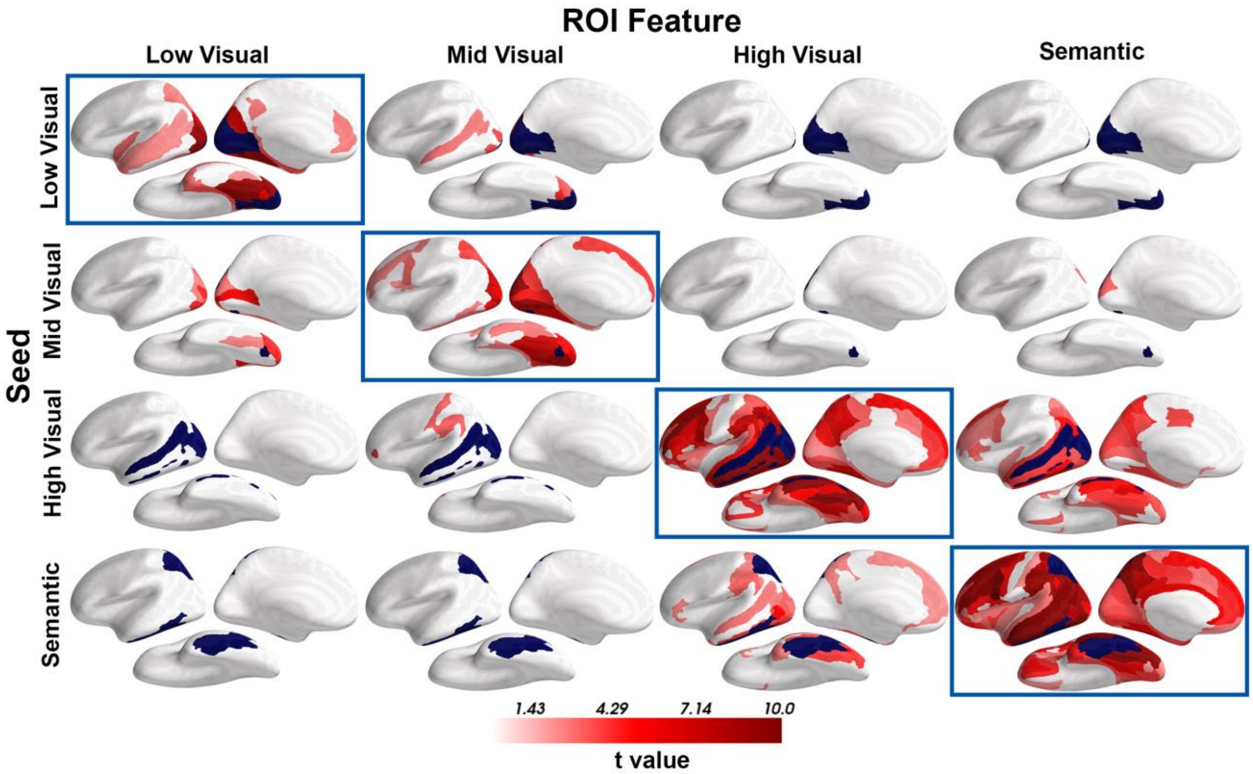

Supplementary Figure 6. Feature-Specific Informational Connectivity during the Recognition Task. A recreation of the analysis depicted in Figure 4b with the exception that the data was drawn from the recognition task (instead of the recall task). The most notable difference between the recognition and recall periods is the lack of low-level FISC within the lateral frontal cortex during recognition. This may be the result of the low-level features being generated in a predominately bottom-up manner during recognition (due to the perception of the probe), and a predominately top-down manner during recall (requiring lateral frontal regions for the construction and/or maintenance of the mental image in the visual cortex).

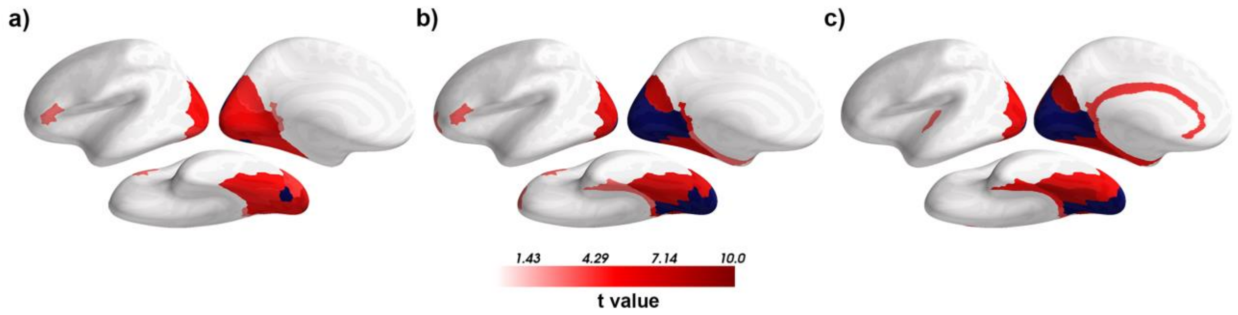

Supplementary Figure 7. Effect of Seed Size on Mid-Level Feature-Specific Informational Connectivity. a) FSIC using the mid-level seed, as depicted in Figure 4. b) FSIC using mid-level reactivation within a combination of the low- and mid-level ROIs (as depicted in Figure 5a: "low + mid") for the seed. Results in a) and b) are nearly identical, indicating that the difference in the extent of low- and mid-level neural reactivation was not due to the relatively small size of the mid-level ROI. c) FSIC using mid-level reactivation within the low-level ROIs for the seed. Despite using the same ROI weights as the low-level seed, the results for mid-level features within the frontal cortex depicted in c) differ greatly from the low-level results depicted in the top-left of figure 4b, providing strong evidence that the findings are not due to noise correlations between regions. t-values are thresholded at $p < 0.05$, one-tailed, FDR corrected.

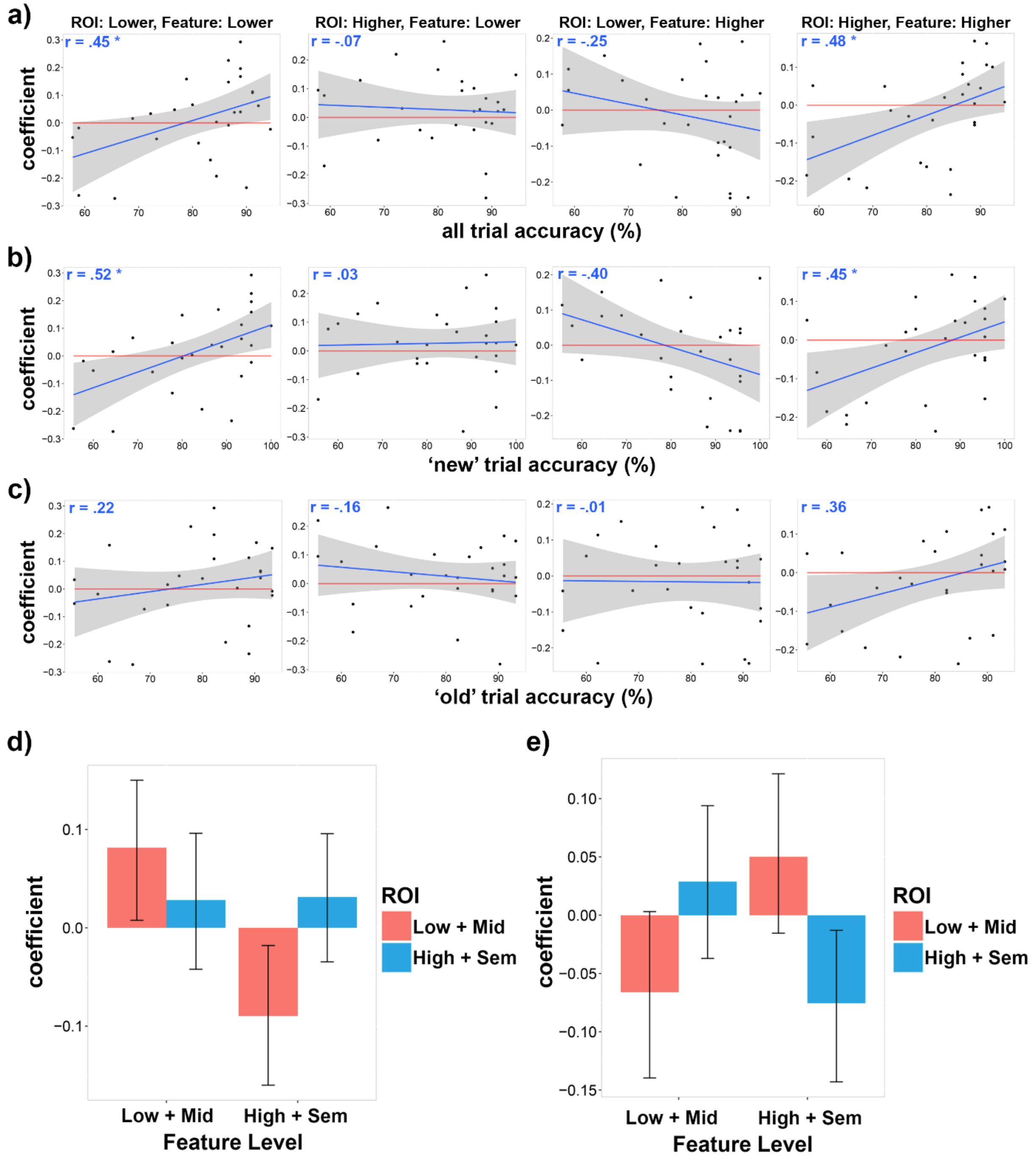

Supplementary Figure 8. Individual Differences in the Correlation Between Recognition Accuracy and Neural Reactivation during Recall. a-c) The between-subject correlation between average old/new task accuracy (percentage correct for a) all trials, b) 'new'/lure trials, and c) 'old' trials) and the within-subject partial correlation coefficient between old/new accuracy and neural reinstatement for all four combinations of ROI and feature-level. Points represent subjects; grey region indicates 95% CI; * indicates $p < .05$, two-tailed t-test, FDR corrected. d-e) Within-subject partial regression coefficients between neural reactivation and

old/new task accuracy for all combinations of feature-level and ROI, with the participants divided into two groups: d) the thirteen subjects with the highest average ‘new’/lure trial accuracy, and e) the thirteen subjects with the lowest average ‘new’/lure trial accuracy. Error bars are 95% CI. Consistent with the between-subject results, reactivation encoding lower-level features positively correlated with accuracy (before considering the other coefficients), but only for the high-lure-accuracy group, i.e. subjects who were less likely to label a similar new image as previously seen. When considering all coefficients, no coefficient was significantly greater than zero after correcting for multiple comparisons (FDR).

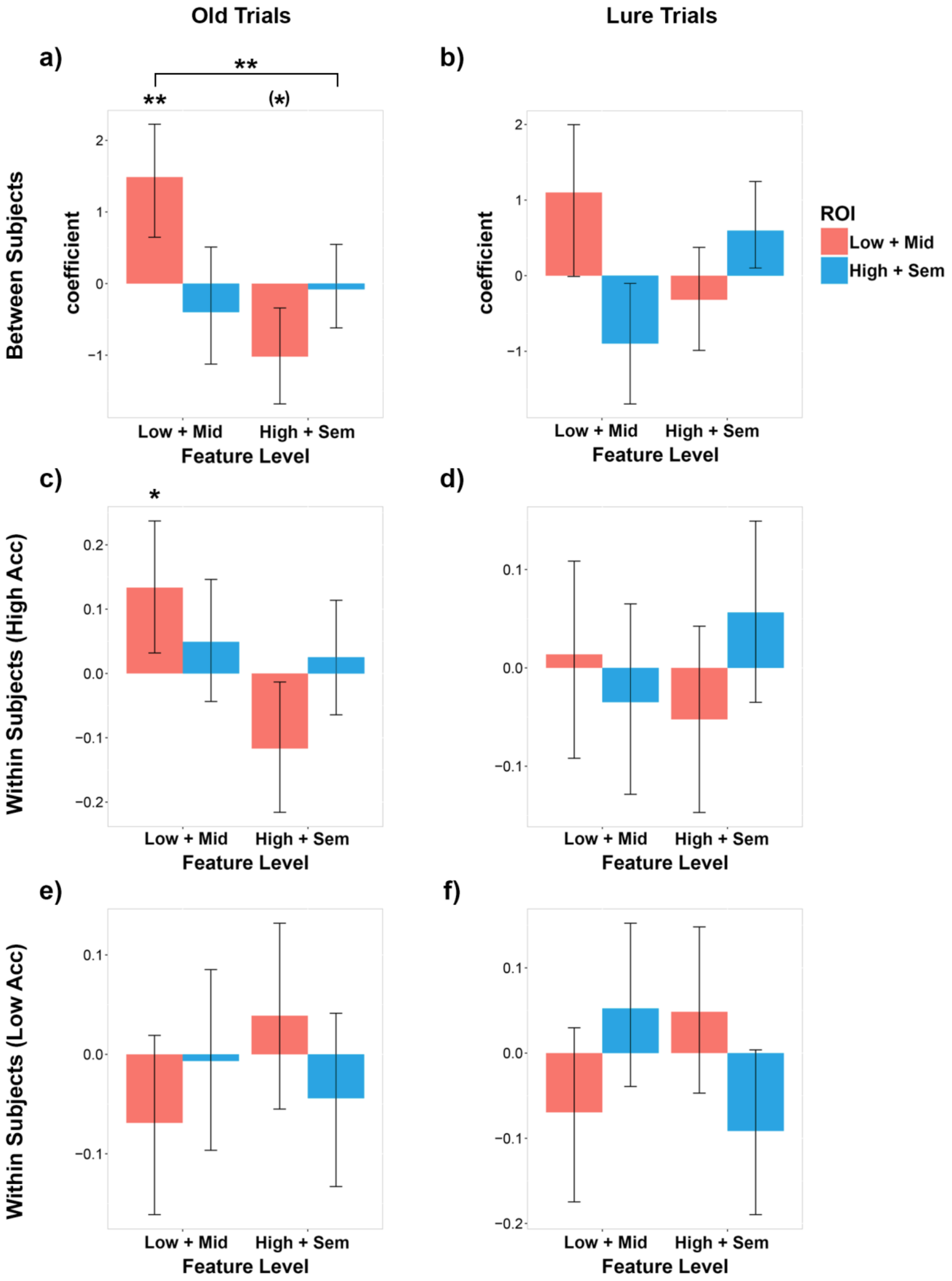

Supplementary Figure 9. Correlations Between Feature-Specific Neural Reactivation and Recognition Accuracy Divided into Old and Lure Trials. a-b) Between-subject partial

regression coefficients measuring the relation between neural reactivation and recognition accuracy (during the old and lure trials, respectively) for all combinations of feature-level and ROI. c-d) Within-subject partial regression coefficients measuring the relation between neural reactivation and recognition accuracy (during the old and lure trials, respectively) for the thirteen subjects with the highest average ‘new’/lure trial accuracy. e-f) Within-subject partial regression coefficients measuring the relation between neural reactivation and recognition accuracy (during the old and lure trials, respectively) for the thirteen subjects with the lowest average ‘new’/lure trial accuracy. The error bars are 95% CIs; * indicates $p < .05$, one-tailed bootstrap; (*) indicates $p < .05$, two-tailed bootstrap; FDR corrected over the four coefficients. For both between- and within-subject analyses, low-level reactivation significantly correlated with old-trial accuracy and not lure-trial accuracy (note: the within-subject correlation between accuracy and vividness was also limited to old trials; see results section “Relation Between Vividness Ratings and Old/New Task Accuracy” and Supplementary Figure 1). Our results are consistent with the idea that the high-lure-accuracy subjects utilize recalled low-level details (e.g. edges) for the recognition task, but only when no difference in high-level/semantic features (between the subject’s memory and the probe) is clearly evident (which would happen more often on old trials, because there are no differences—assuming an accurate memory).

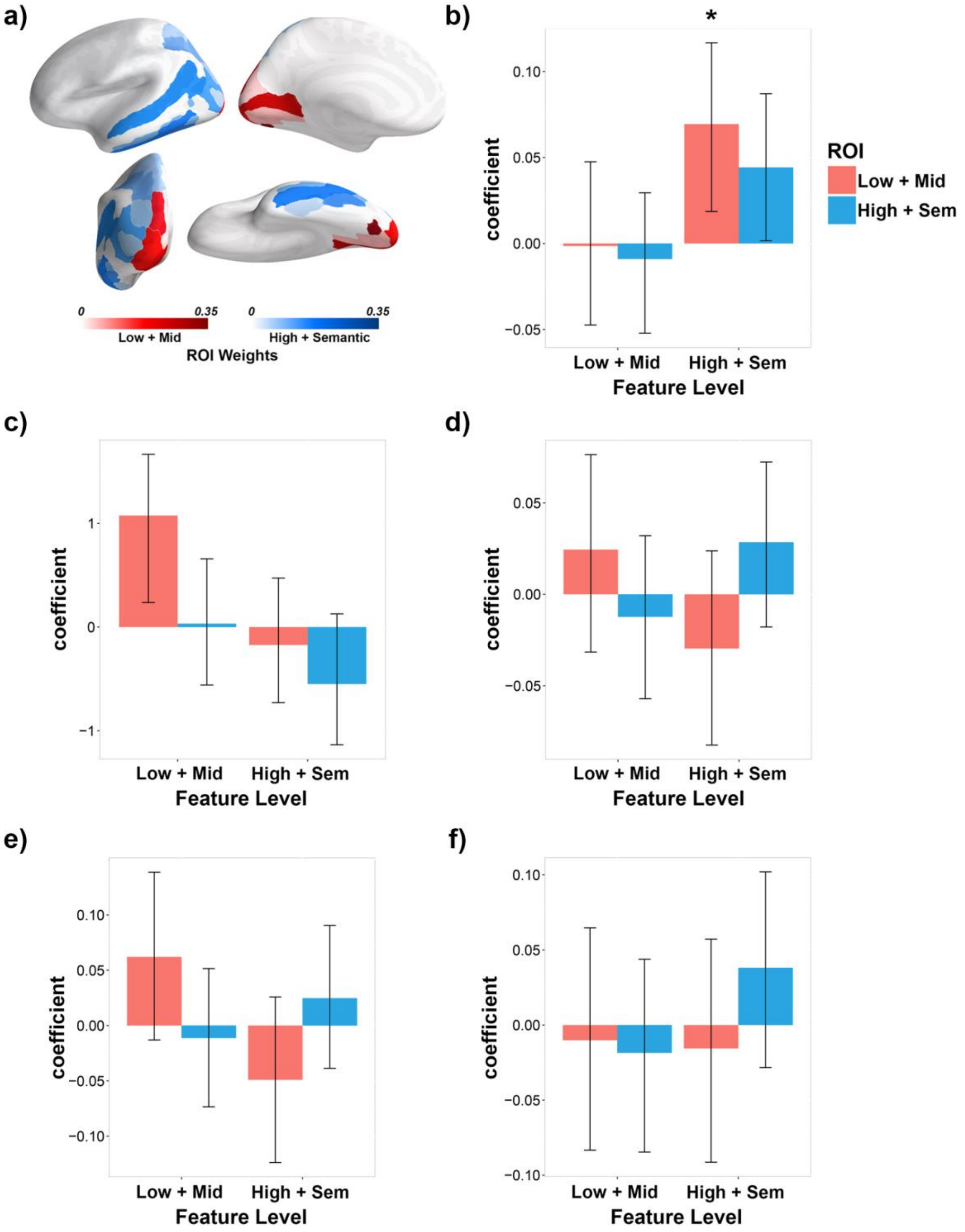

Supplementary Figure 10. Correlations Between Feature-Specific Neural Reactivation During the Recognition Task, Vividness and Recognition Accuracy. a) ROI weights combining the low- and mid-level and high- and semantic-level ROIs. b) Within-subject partial regression coefficients measuring the relation between neural reactivation during recognition (when the recognition probe was presented on the screen) and vividness for all combinations of

feature-level and ROI. c) Between-subject partial regression coefficients measuring the relation between neural reactivation during the recognition task and recognition accuracy for all combinations of feature-level and ROI. d) Within-subject partial regression coefficients measuring the relation between neural reactivation during the recognition task and recognition accuracy for all combinations of feature-level and ROI. e-f) Within-subject partial regression coefficients between neural reactivation and old/new task accuracy for all combinations of feature-level and ROI, with the participants divided into two groups: d) the thirteen subjects with the highest average 'new'/lure trial accuracy, and e) the thirteen subjects with the lowest average 'new'/lure trial accuracy. The error bars are 95% CIs; * indicates $p < .05$, two-tailed bootstrap, FDR corrected over the four coefficients. Caution should be taken in interpreting these results because patterns of neural activation caused by perception of the recognition probe would be conflated with (and consequently obscure) memory-driven neural reactivation—particularly for low-level features within the early visual cortex. Our main findings used reactivation during the recall period to avoid this issue.

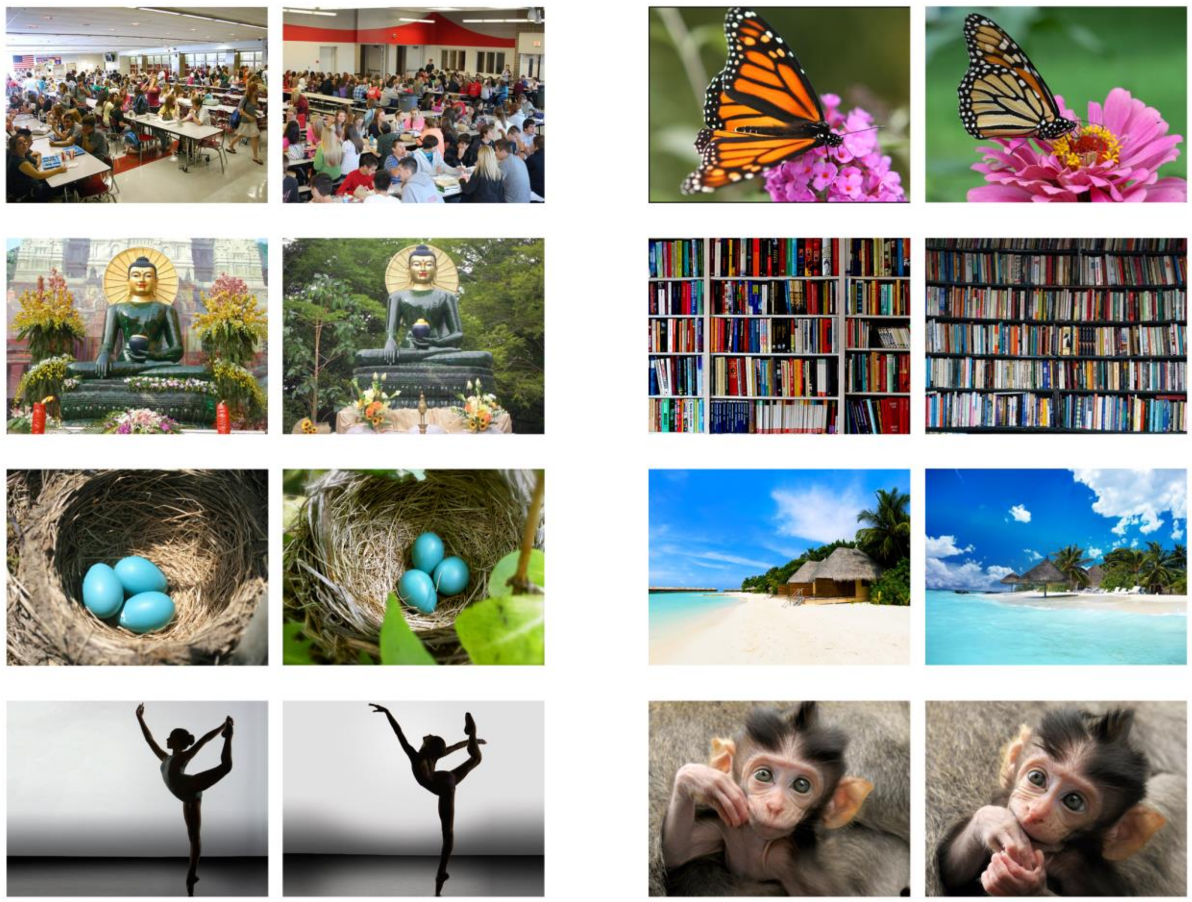

Supplementary Figure 11. Example of Image Pairs. Eight randomly selected image pairs out of the ninety total.

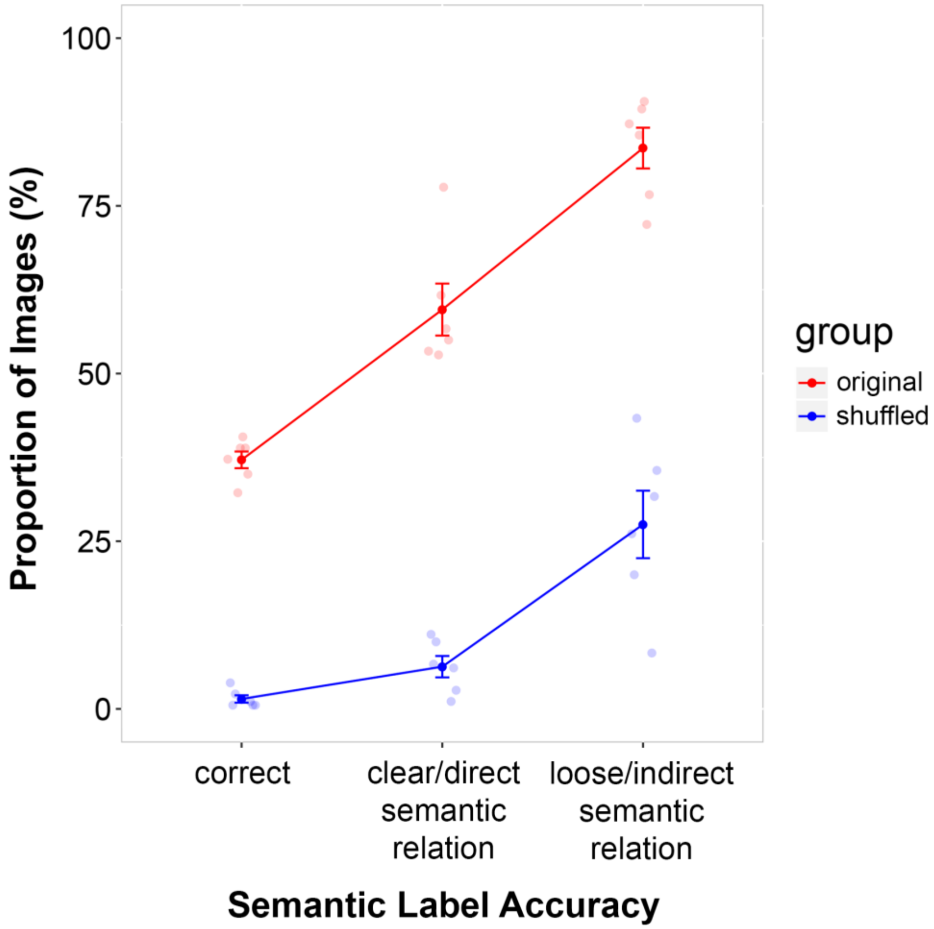

Supplementary Figure 12. VGG16 Top 5 Semantic Classification Accuracy for the 180 Encoding/Retrieval images. Twelve participants (not including any participants from the main study) rated the accuracy of the top 5 (out of 1000) semantic labels predicted by VGG16. For a null reference, the labels were randomly shuffled (over the 180 images) for six subjects (in blue). There were four possible ratings: 1) correct classification, i.e. at least one of the labels was in the image (we were interested in general rather than specific semantic categories, e.g. if the image contained a dog then any dog breed label would be considered to be in the image); 2) at least one of the labels had a clear/direct semantic relation to the image; 3) at least one of the labels had a loose/indirect semantic relation to the image; 4) none of the labels had any semantic relation to the image. The proportions indicated in the graph are cumulative from left to right. Mean proportions are displayed opaquely. Proportions for individual participants are displayed semi transparently. Error bars are ±1 SEM.

Supplementary Notes

Supplementary Note 1

The mean vividness rating over trials, averaged across participants, was 3.04 (SD = 0.35). On average, 2.9% of trials were rated as vividness = 1, 19.7% as vividness = 2, 48.0% as vividness = 3 and 29.4% as vividness = 4. Participants failed to respond within the three second vividness rating period on 0.9% (SD = 2.2%) of the trials. These trials were excluded from all analyses.

To investigate whether vividness ratings were consistent across participants, the participants' vividness values for all 90 recalled images were correlated with the average vividness values across all other subjects for those images (only using those subjects that saw the target image during encoding, as opposed to its pair). The mean correlation value across subjects was significantly greater than zero [$r = .18$, $p < .005$, one-tailed 200 sample permutation test], indicating that participants consistently rated some images as more vivid than others (we accounted for this variation in the average recalled vividness of different items by including 'image' as a random effect in all applicable linear mixed models).

In order to validate the subjective vividness ratings, we set out to determine if trials with higher ratings were associated with higher recognition accuracy. The relation between vividness ratings and accuracy (see the "Relation Between Vividness Ratings and Old/New Task Accuracy" section, and Supplementary Figure 1) was assessed using three LME models (one for all trials (n = 2406) , one limited to old trials (n = 1190) and one limited to lure trials (n = 1216)), with accuracy (correct = 1, incorrect = 0) as the dependent variable (DV), vividness rating (1-to-4) as the independent variable (IV), and participant and image label as crossed random effects (random-intercept only, due to model complexity limitations). Confidence intervals and p-values

were calculated with bootstrap statistical analyses using the BootMer function[1]. A significant positive relation between vividness and accuracy was found when all trials ("old" and "new"/lure) were included in the analysis [$\beta = .036$, $p < .001$, one-tailed 1000 sample bootstrap]. Dividing the trials into "old" and "new"/lure conditions, a significant positive relation was found for "old" trials [$\beta = .058$, $p < .001$, one-tailed 1000 sample bootstrap], whereas "new"/lure trials showed a marginal positive relation [$\beta = .021$, $p = .088$, one-tailed 1000 sample bootstrap]. See Supplementary Figure 1 for more detail.

Supplementary Note 2

The means for old/new task accuracy and confidence ratings, averaged across participants, were 81.0% (SD = 11.0%; chance = 50%) and 3.46 (SD = 0.30), respectively. Accuracy on "old" and "new"/lure trials was 79.2% (SD = 12.3%) and 82.8% (SD = 13.4%), respectively, with no significant difference in accuracy between the two conditions ($t(26) = 1.34$, $p = .193$, paired samples, two-tailed t-test). On average, 3.1% of trials were rated as confidence = 1, 10.9% as confidence = 2, 22.1% as confidence = 3 and 63.9% as confidence = 4. The association between accuracy and confidence ratings was significant ($\beta = .89$, $p < .001$, two-tailed 100 sample bootrap) (measured using a generalized linear mixed-effects (LME) model with subject and image as crossed random effects). Participants failed to respond within the three second old/new response period on 1.0% (SD = 1.5%) of the trials, and the two second confidence rating period on 1.8% (SD = 2.3%) of the trials. The former trials were classified as incorrect, while the latter were excluded from analyses that incorporated confidence ratings.

Supplementary Note 3

As with any model of feature-specific cortical representations, the features extracted from VGG16 (the CNN used in this study) cannot be expected to be a complete set of all visual

features represented within a given participant's cortical activity. Consequently, our approach cannot exhaustively control for all inter-level correlations, potentially resulting in the false detection of feature-specific neural reactivation. To address this concern, consider two possibilities: 1) that lower-level features tend to be detected within regions that only contain higher-level features, and 2) that higher-level features tend to be detected within regions that only contain lower-level features. If the former was true, we would expect approximately equal reactivation of mid-level features relative to low-level features within higher-order cortical regions, due to the mid-level and low-level features correlating with higher-level features to approximately the same degree (Supplementary Figure 3). In contrast, low-level reactivation was much more pronounced than mid-level reactivation (Figure 4b; first two rows along the diagonal). If the latter was true, then higher-level features would be expected within the earliest region of the visual cortex: the calcarine sulcus. This was not the case (Figure 4b: bottom right).

Supplementary Note 4

Suppose a study participant is recalling one of two images per trial and 50% of the time the subject accurately recalls the target image, while 50% of the time the subject mistakenly recalls the other (non-target) image. Let us further assume that image decoding during recall is 100% accurate. In this case, the mean decoding accuracy (for the target image) would be at chance (50%) leading to the false conclusion that the features being decoded are not present in the ROI during recall. In contrast, if we correlated trial-by-trial decoding accuracy between two regions (each with 100% decoding accuracy) we would get a perfect correlation, correctly indicating that the relevant features are represented in the ROIs. More realistically, any variance in recall accuracy (i.e. deviation from perfect recall) would reduce the power of studies using mean decoding accuracy, while not adversely affecting the power of studies using the trial-by-trial

correlation method, i.e. FSIC.

Supplementary Note 5

According to the predictive coding account of perception, top-down connections from neurons that encode high-level/semantic features drive neural activity representing lower-level features to generate a model of the expected stimulus, which is compared against the perceptual input to generate an error signal[2-5]. From this perspective, the presence of higher-level features within the lower-level ROI may represent the top-down inference of lower-level features because these inferred features would be yoked to the associated high-level features. When reactivation of the perceived low-level features is statistically controlled for—as in the above analysis—the reactivation of higher-level features within the lower-level ROI is constrained to represent only the *incorrect* inferences, i.e. predictions of low-level features that were not present in the encoded image. These incorrect inferences would result in mental imagery of a generic image associated with the recalled/cued high-level/semantic features which participants were instructed not to rate as vivid—even if the generic mental image contained many visual details. Therefore, the observed negative partial correlation between vividness and neural reactivation of higher-level features within the lower-level ROI is consistent with a predictive coding account of perception and memory recall.

Supplementary References

1. Bates, D., Maechler, M., Bolker, B., Walker, S. Fitting linear mixed-effects models using lme4. Journal of Statistical Software. 67:1-48 (2015).

2. Rao, R. P., & Ballard, D. H. (1999). Predictive coding in the visual cortex: a functional

interpretation of some extra-classical receptive-field effects. Nature neuroscience, 2(1), 79.

3. Friston, K. (2005). A theory of cortical responses. Philosophical Transactions of the Royal Society of London B: Biological Sciences, 360(1456), 815-836.

4. Friston, K. (2010). The free-energy principle: a unified brain theory?. Nature reviews neuroscience, 11(2), 127.

5. Bastos, A. M., Usrey, W. M., Adams, R. A., Mangun, G. R., Fries, P., & Friston, K. J. (2012). Canonical microcircuits for predictive coding. Neuron, 76(4), 695-711.
