# Supplementary material for: Feature-specific neural reactivation during episodic memory
Source: Nat Commun. 2020 Apr 23;11:1945. doi: 10.1038/s41467-020-15763-2 (PMC7181630; doi:10.1038/s41467-020-15763-2)
Supplement: Supplementary file 3 — Reporting Summary [file 41467_2020_15763_MOESM3_ESM.pdf]

nature research

Corresponding author(s): Michael B Bone

Last updated by author(s): Jan 30, 2020

Reporting Summary

Nature Research wishes to improve the reproducibility of the work that we publish. This form provides structure for consistency and transparency in reporting. For further information on Nature Research policies, see Authors & Referees and the Editorial Policy Checklist.

Statistics

For all statistical analyses, confirm that the following items are present in the figure legend, table legend, main text, or Methods section.

n/a	Confirmed	
☐	☒	The exact sample size (*n*) for each experimental group/condition, given as a discrete number and unit of measurement
☐	☒	A statement on whether measurements were taken from distinct samples or whether the same sample was measured repeatedly
☐	☒	The statistical test(s) used AND whether they are one- or two-sided<br>*Only common tests should be described solely by name; describe more complex techniques in the Methods section.*
☐	☒	A description of all covariates tested
☐	☒	A description of any assumptions or corrections, such as tests of normality and adjustment for multiple comparisons
☐	☒	A full description of the statistical parameters including central tendency (e.g. means) or other basic estimates (e.g. regression coefficient) AND variation (e.g. standard deviation) or associated estimates of uncertainty (e.g. confidence intervals)
☐	☒	For null hypothesis testing, the test statistic (e.g. *F*, *t*, *r*) with confidence intervals, effect sizes, degrees of freedom and *P* value noted<br>*Give P values as exact values whenever suitable.*
☒	☐	For Bayesian analysis, information on the choice of priors and Markov chain Monte Carlo settings
☐	☒	For hierarchical and complex designs, identification of the appropriate level for tests and full reporting of outcomes
☐	☒	Estimates of effect sizes (e.g. Cohen's *d*, Pearson's *r*), indicating how they were calculated

Our web collection on statistics for biologists contains articles on many of the points above.

Software and code

Policy information about availability of computer code

Data collection	E-Prime 2.0.10.353<br>Commercial code bundled with the Siemens Tim Trio system for acquisition and reconstruction of MRI images.
Data analysis	CNN features:<br>features extracted from VGG16 implementation in TensorFlow version 1.13.1 (http://www.cs.toronto.edu/~frossard/post/vgg16/)<br><br>Encoding-Decoding model and Feature-Specific Neural Reactivation:<br>custom code programmed in R version 3.5.3<br>nnlasso package version 0.3 was used to generate the parameters of the encoding model<br><br>For statistical analyses:<br>programmed in R version 3.5.3<br>lme4 package version 1.1-21 used for LME models

For manuscripts utilizing custom algorithms or software that are central to the research but not yet described in published literature, software must be made available to editors/reviewers. We strongly encourage code deposition in a community repository (e.g. GitHub). See the Nature Research guidelines for submitting code & software for further information.

Data

Policy information about availability of data

All manuscripts must include a data availability statement. This statement should provide the following information, where applicable:

- Accession codes, unique identifiers, or web links for publicly available datasets
- A list of figures that have associated raw data
- A description of any restrictions on data availability

Data for all analyses covered in the article is available at https://github.com/MichaelBBone/FSIC-During-Episodic-Memory/releases.

Field-specific reporting

Please select the one below that is the best fit for your research. If you are not sure, read the appropriate sections before making your selection.

☒ Life sciences ☐ Behavioural & social sciences ☐ Ecological, evolutionary & environmental sciences

For a reference copy of the document with all sections, see nature.com/documents/nr-reporting-summary-flat.pdf

Life sciences study design

All studies must disclose on these points even when the disclosure is negative.

Sample size	Data from 37 participants was collected. Data from 27 participants was included in the study (15 males and 12 females, 20-32 years old [mean: 25]).<br>A sample of 27 offers power to detect medium-to-large directional effects at 80% power.
Data exclusions	Data from ten participants (out of the original 37) were excluded from the final analyses for the following reasons: excessive head motion (5; removed if > 5mm within run maximum displacement in head motion), fell asleep (2), did not complete experiment (3).
Replication	There was no attempt at replication.
Randomization	There were no experimental groups.
Blinding	There was no blinding to group allocation, because there was no group allocation relevant to the outcome of the study.

Reporting for specific materials, systems and methods

We require information from authors about some types of materials, experimental systems and methods used in many studies. Here, indicate whether each material, system or method listed is relevant to your study. If you are not sure if a list item applies to your research, read the appropriate section before selecting a response.

Materials & experimental systems

n/a	Involved in the study	
☒	☐	Antibodies
☒	☐	Eukaryotic cell lines
☒	☐	Palaeontology
☒	☐	Animals and other organisms
☐	☒	Human research participants
☒	☐	Clinical data

Methods

n/a	Involved in the study	
☒	☐	ChIP-seq
☒	☐	Flow cytometry
☐	☒	MRI-based neuroimaging

Human research participants

Policy information about studies involving human research participants

Population characteristics	27 participants were included in the study (15 males and 12 females, 20-32 years old [mean: 25]). Participants had normal or corrected-to-normal vision and no history of neurological or psychiatric disease. Participants were either native or fluent English speakers and had no contraindications for MRI.
Recruitment	Participants were recruited through the Baycrest subject pool and paid for their participation. There is no sampling bias that we believe to be relevant to the current study other than age. Young adults were recruited to avoid age-related memory degradation.
Ethics oversight	The study was approved by the Rotman Research Institute's Ethics Board.

Note that full information on the approval of the study protocol must also be provided in the manuscript.

Magnetic resonance imaging

Experimental design

Design type	task-based block design
Design specifications	3 video viewing runs, each 10m 57s seconds long.<br><br>3 encoding runs, each 6m 24s long. 120 trials per run, with 1.8s image presentation and 1.7s ISI per trial.<br><br>3 retrieval runs, each 9m 32s long. 30 trials per run, with 1s cue, 6s recall, 3s vividness rating, 3s recognition task and 3s confidence rating per trial.
Behavioral performance measures	button presses using four-button fiber optic response box.<br><br>Encoding 1-back task: 1 if the displayed image was the same as the preceding image, and 2 otherwise<br><br>Vividness rating: 1-4 scale (1 = low, 4 = high)<br>Recognition task: 1 if they thought that the image was the one seen during encoding (old), or 2 if the image was new<br>Confidence rating: 1-4 scale (1 = low, 4 = high)<br><br>The mean, proportion and standard deviation of retrieval responses were assessed.

Acquisition

Imaging type(s)	functional
Field strength	3T
Sequence & imaging parameters	A high-resolution gradient-echo multi-slice T1-weighted scan coplanar with the echo-planar imaging scans (EPIs) was first acquired for localization. Functional images were acquired using a multiband EPI sequence sensitive to BOLD contrast (22 x 22 cm field of view with a 110 x 110 matrix size, resulting in an in-plane resolution of 2 x 2 mm for each of 63 2-mm axial slices; repetition time = 1.77 sec; echo time = 30ms; flip angle = 62 degrees). A high-resolution whole-brain magnetization prepared rapid gradient echo (MP-RAGE) 3-D T1 weighted scan (160 slices of 1mm thickness, 19.2 x 25.6 cm field of view) was also acquired for anatomical localization.
Area of acquisition	whole brain

Diffusion MRI ☐ Used ☒ Not used

Preprocessing

Preprocessing software	AFNI version 18.0.22 for motion correction with 3dVolreg and co-registration using 3dAllineate via the align_epi_anat.py script
Normalization	Freesurfer version 5.3 for surface extraction and spherical normalization
Normalization template	fsaverage distributed with Freesurfer 5.3
Noise and artifact removal	structured noise removed via high-pass filter using polynomial basis functions as implemented in AFNI's 3dDeconvolve
Volume censoring	no volume censoring was used

Statistical modeling & inference

Model type and settings	A multivariate encoding approach was used to predict multi-vertex activity patterns during the 6s recall period for each retrieval trial. An encoding model was estimated for each of the four feature levels and each individual vertex. For the model, non-negative lasso regression (R package "nnlasso") was used, with the image features as independent variables and vertex activity as the dependent variable.
Effect(s) tested	The correlation between model predictions and the activity measured during visual recall was used to decode the cued image for each feature level, thereby measuring neural reactivation.<br><br>To measure feature-specific neural reactivation it was necessary to control for inter-feature-level correlations.<br><br>For determining the cortical distribution of feature-specific neural reactivation, we used a new measure, feature-specific informational connectivity (FSIC), which is the partial correlation of decoding accuracy for a given feature-level across ROIs, controlling for the decoding accuracy of all non-target feature-levels. The correlations were calculated with a linear mixed-effects (LME) model on data from all episodic recall trials, wherein classification accuracy for the seed ROI was the dependent variable, classification accuracy for each of the four feature levels within the target ROI were

the independent variables, and participant and image were crossed random effects (random-intercept only, due to model complexity limitations).

For determining the within-subject correlations between feature-specific neural reactivation, vividness ratings, and recognition accuracy, LME models were used, with vividness ratings or recognition accuracy (correct vs incorrect) as the dependent variable, decoding accuracy for each combination of ROI (lower-level and higher-level) and feature-level (lower-level and higher-level) as independent variables, and participant and image as crossed random effects (random-intercept only, due to model complexity limitations).

For the between-subject partial correlations between feature-specific reactivation and recognition accuracy, a single linear model was used, with mean recognition accuracy as the dependent variable and mean decoding accuracy for each combination of ROI (lower-level and higher-level) and feature-level (lower-level and higher-level) as independent variables.

Specify type of analysis: ☐ Whole brain ☒ ROI-based ☐ Both

Anatomical location(s): 148 cortical Freesurfer ROIs (Destrieux, Fischl, Dale, & Halgren, 2010)

Statistic type for inference (See Eklund et al. 2016): See 'effects tested' above.

Correction: False discovery rate (FDR) was used to correct for multiple comparisons across partial correlation coefficients for our behavioral results and across ROIs for all other results.

Models & analysis

n/a	Involved in the study
☒	☐ Functional and/or effective connectivity
☒	☐ Graph analysis
☐	☒ Multivariate modeling or predictive analysis

Multivariate modeling and predictive analysis: A multivariate encoding approach was used to predict multi-vertex activity patterns during the 6s recall period for each retrieval trial. An encoding model was estimated for each of the four feature levels and each individual vertex. For the model, non-negative lasso regression (R package "nnlasso") was used, with the image features as independent variables and vertex activity as the dependent variable.

The correlation between model predictions and the activity measured during visual recall was used to decode the cued image for each feature level, thereby measuring neural reactivation. The accuracy of this prediction was assessed as follows: 1) for each combination of subject, feature-level, and ROI the predicted neural activation patterns for the 90 images viewed during the encoding task were generated using a model that was trained on the movie and encoding task data, excluding data from encoding trials wherein the predicted image was viewed using 3-fold cross validation. 2) for each retrieval trial, the predictions were correlated (across vertices within the given ROI) with the observed neural activity during recall resulting in 90 correlation values (r). 3) the correlation values were ranked in descending order, and the rank of the prediction associated with the recalled image was recorded (1 = highest accuracy, 90 = lowest accuracy). 4) this rank was then subtracted from the mean rank (45.5) so that 0 was chance, and a positive value indicated greater-than-chance accuracy for the given trial (44.5 = highest accuracy, -44.5 = lowest accuracy).
